# Trends of Antimicrobial Resistance in Typhoidal Strains of Salmonella in a Tertiary Care Hospital in Pakistan

**DOI:** 10.7759/cureus.12664

**Published:** 2021-01-12

**Authors:** Aqsa Aslam, Sahibzada Ahmed Kharal, Maria Aslam, Almas Raza

**Affiliations:** 1 Microbiology, National University of Medical Sciences, Rawalpindi, PAK; 2 Family Medicine, Forrest Family Practice, Bunbury, AUS; 3 Hematology, Sharif Medical and Dental College, Lahore, PAK; 4 Microbiology, Pak Red Crescent Medical and Dental College, Lahore, PAK

**Keywords:** typhoidal strains of salmonella, antimicrobial resistance, salmonella typhi, salmonella paratyphi

## Abstract

Introduction: Enteric fever or typhoid fever is a major public health issue affecting greater than 27 million individuals globally and is responsible for greater than 200,000 deaths per year. Due to the extensive overuse of antimicrobials, the world is moving toward a pre-antibiotic era. The emergence and transmission of antibiotic-resistant *Salmonella* species are a global threat and a serious concern in developing countries such as Pakistan. This study aimed to determine the trends in antimicrobial resistance (AMR) of typhoidal strains of *Salmonella* in a tertiary care hospital in Pakistan.

Materials and Methods: It was a descriptive, cross-sectional study conducted in the pathology department of Sharif City Hospital, Lahore, after approval by the ethical committee of the institution. A total of 50 blood culture specimens positive for *Salmonella typhi* and *Salmonella paratyphi* from January 2019 to March 2020 were included by the non-probability consecutive sampling technique. The samples were processed by conventional bacteriological methods for isolation and identification. The antimicrobial susceptibility testing was done by the Kirby-Bauer disc diffusion method as recommended by the Clinical and Laboratory Standard Institute (CLSI). The statistical package for social sciences (SPSS, IBM Corp., Armonk, NY) version 25 was used for data entry and analysis.

Results: Among the first-line drugs (ampicillin, chloramphenicol, and trimethoprim-sulfamethoxazole), 70% of strains were resistant, and only 30% strains were sensitive to them. Among the cephalosporins, 52% strains were sensitive to ceftriaxone, and 48% strains were sensitive to ceftazidime, cefotaxime, and cefepime. Twenty-four percent of strains were sensitive to ciprofloxacin. Only 50% of strains were sensitive to ampicillin-sulbactam, and 92% of strains were sensitive to piperacillin-tazobactam. All the strains were 100% sensitive to imipenem and meropenem; 96% of strains were sensitive to co-amoxiclav, doxycycline, and azithromycin. The frequency of multidrug-resistant (MDR) and extensively drug-resistant (XDR) *Salmonella* species was 16% and 54%.

Conclusion: The increasing frequency of MDR and XDR *Salmonella* species in Pakistan is a major concern. A significant percentage of the typhoidal strains of *Salmonella* is resistant to the first-line (16%) and second-line (54%) antibiotics. Carbapenems and azithromycin are the last resort of therapy in such cases.

## Introduction

Enteric fever or typhoid fever is an acute, fatal febrile illness with a mortality rate of 1% to 3%. It is a major public health issue affecting greater than 27 million individuals globally and is responsible for greater than 200,000 deaths per year [1,2]. It causes significant morbidity and mortality globally, especially in developing countries. Its causative organisms are *Salmonella typhi *and *Salmonella paratyphi A, B*, and *C* [3]. The transmission is through the fecal-oral route mainly through contaminated water. Patients with enteric fever present with high-grade fever, malaise, headache, anorexia, nausea, abdominal pain, and diarrhea or constipation. Physical examination reveals hepatomegaly, splenomegaly, and relative bradycardia. The complications of this infection are intestinal perforation, bleeding, peritonitis, and meningoencephalitis. The diagnostic test for enteric fever is blood culture or bone marrow culture in the first week [1-4]. The mainstay of treatment is antibiotics. The first-line antibiotics are ampicillin, chloramphenicol, and trimethoprim-sulfamethoxazole. The second-line antibiotics are fluoroquinolones. Ceftriaxone, azithromycin, and carbapenems are used when isolates show resistance to first- and second-line antibiotics. The effective ways for the prevention of enteric fever are vaccination, hand hygiene, improved sanitation, and the use of clean water [5].

The genus *Salmonella* is a facultative anaerobe, gram-negative bacillus, and is classified into two species, *Salmonella enterica *and *Salmonella bongori* [1]. It is a member of the Enterobacteriaceae family [2]. *Salmonella enterica *subspecies *enterica *has 2,600 serovars, out of which four serovars are clinically important. *Salmonella typhi* and *Salmonella paratyphi* cause enteric fever, while *Salmonella typhimurium* and *Salmonella enteritidis* cause non-typhoidal salmonellosis [1,2].

Salmonellosis is common in the developing world including Asia, Africa, and South America [6]. According to a study, enteric fever has a prevalence of 29.3 per 100,000 person-years in China, 24.2 in Vietnam, 180.3 in Indonesia, 493.5 in India, and 412.9 in Pakistan [3]. Multidrug-resistant* Salmonella* has been reported from South Asia. Multidrug-resistant *Salmonella* is strain-resistant to first-line antibiotics (ampicillin, chloramphenicol, and trimethoprim-sulfamethoxazole) [6]. Nowadays, extensively drug-resistant (XDR) *Salmonella* has emerged as an important health issue in Pakistan. These isolates can spread globally via conjugation or transposons and transform MDR strains to XDR strains. *Salmonella* strains are labeled as XDR when they show resistance to first-line antibiotics, fluoroquinolones, and third-generation cephalosporins [7]. Extensively drug-resistant *Salmonella *was first reported in 2016 in Hyderabad, Sindh [8]. The analysis of the genome sequence of these strains showed that they had an H58 haplotype [6]. The treatment of choice for these strains is azithromycin and meropenem. Azithromycin resistance has also been documented in India [9]. The antibiotic resistance genes (AMR) in the H58 haplotype are associated with an IncHI1 plasmid. These genes include blaTEM-1 conferring resistance to ampicillin, dfrA7, sul1, sul2-mediating resistance to trimethoprim-sulfamethoxazole, and catA1 causing chloramphenicol resistance. Ceftriaxone resistance is conferred by the extended-spectrum β lactamase (ESBL) gene. Resistance to fluoroquinolones is caused by mutations in the DNA gyrase (gyrA and gyrB) and topoisomerase IV genes [10].

The World Health Organization (WHO) reported 5274 cases of XDR *Salmonella typhi* cases in Sindh from 2016 to 2018. Seventy-six percent of these cases were from Karachi city followed by Hyderabad (27%) and other Sindh districts (4%) [6,11]. Instead of the awareness campaigns and infection control measures implemented by the government, the frequency of XDR *Salmonella typhi *cases raised from 2017 to 2018 [7]. Five cases of XDR *Salmonella typhi *were reported from the United States, one case from the United Kingdom, and one case from Canada. But all of them traveled to Pakistan [12,13]. The study aimed to determine the trends in antimicrobial resistance (AMR) of typhoidal strains of *Salmonella *in a tertiary care hospital in Pakistan. Due to the extensive overuse of antimicrobials, the world is moving toward a pre-antibiotic era. The emergence and transmission of antibiotic-resistant *Salmonella *species are a global threat and a serious concern in developing countries such as Pakistan. Our study will add data and set a priority for future research in this aspect. This study will help in the scaling-up of policies at the government level in Pakistan and other countries with a high prevalence of disease to combat the rising burden of enteric fever. In addition, antimicrobial surveillance is poor, and antibiotic stewardship policies should be implemented.

## Materials and methods

It was a descriptive, cross-sectional study done in the Pathology Department of Sharif City Hospital, Lahore, after approval by the ethical committee of the institution. A total of 1510 blood culture specimens positive for *Salmonella typhi *and *Salmonella paratyphi *from January 2019 to March 2020 were included by the non-probability consecutive sampling technique.

The samples for blood culture were received in the microbiology section of the laboratory in blood culture bottles containing tryptic soya broth and polyanethol sulfonate. The bottles were placed in the incubator at 35°C for five days. The first subculture was performed on blood agar and MacConkey agar after 48 hours. After the first culture was negative, the second subculture was inoculated on blood agar and MacConkey agar in the following 72 hours. *Salmonella *species* *produce non-lactose fermenting colonies on MacConkey agar and were identified by colony morphology, gram staining, and oxidase test. In the case of gram-negative rods and negative oxidase test, they were further identified at the species level using the analytical profile index (API) and slide agglutination test with antisera. Direct gram staining from blood culture bottles and reculture of specimens were also done to confirm the presence of the causative organism. The antibiotic susceptibility testing of the causative organisms was performed using the Kirby-Bauer disc diffusion method. The suspension of the causative organism was made by mixing three to four colonies of the organism in the normal saline and was matched with the 0.5 McFarland turbidity standard. The suspension was evenly applied on the Mueller-Hinton agar plates using the sterile swab, and antibiotic disks were placed on the plates with an aseptic technique. The plates were incubated for 18-24 hours at 35°C, and the zone diameters of antibiotics were evaluated according to the Clinical and Laboratory Standards Institute (CLSI) 2019 [14]. The antibiotics were reported into these categories: susceptible, resistant, or intermediate. The antibiotic panel applied for *Salmonella* included ampicillin (AMP), trimethoprim-sulfamethoxazole (SXT), chloramphenicol (C), ciprofloxacin (CIP), ceftazidime (CAZ), cefotaxime (CTX), ceftriaxone (CRO), cefepime (FEP), imipenem (IPM), meropenem (MEM), piperacillin-tazobactam (TZP), ampicillin-sulbactam (SAM), co-amoxiclav (AMC), doxycycline (DO), and azithromycin (AZM).

The Statistical Package for the Social Sciences (SPSS, IBM Corp., Armonk, New York) software version 25.0 was used for data analysis. The age of the patients was expressed as mean and standard deviation. The gender, respective department of patients, the prevalence of positive blood cultures, and antibiotic susceptibility pattern of the isolates were expressed as frequency and percentages.

## Results

**Figure 1:**
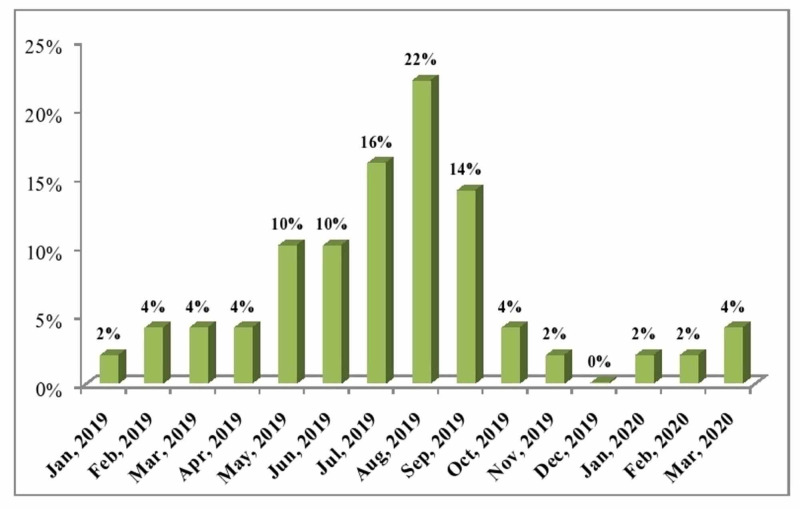
Frequency of Salmonella-Positive Cultures in the 15-Month Period

A total of 1510 blood specimens were received in the pathology laboratory, out of which 50 (3.31%) blood cultures were positive for *Salmonella*. Out of the 50 positive cultures, 47 (94%) were *Salmonella typhi*, and three (6%) were *Salmonella paratyphi*. Among the patients with positive cultures, 34 (68%) were males, while 16 (32%) were females. The majority (60%) of the patients with positive cultures were children ranging from one to 10 years old followed by 11 to 20 years (26%) and 21 to 30 years of age group (8%). Four percent of patients with positive cultures were below one year old and 2% were above 30 years. The distribution of the positive cultures received from different departments of the hospital includes 26 (52%) from the pediatrics department, 11 (22%) from the various outpatient departments, eight (16%) from the medicine department, and five (10%) from the emergency department. Thirty-one (62%) positive cases were reported from June 2019 to September 2019, nine (18%) cases from March to May 2019, and five (10%) cases from October 2019 to February 2020. The frequency of *Salmonella*-positive cultures during the study duration is shown in Figure 1.

**Figure 2:**
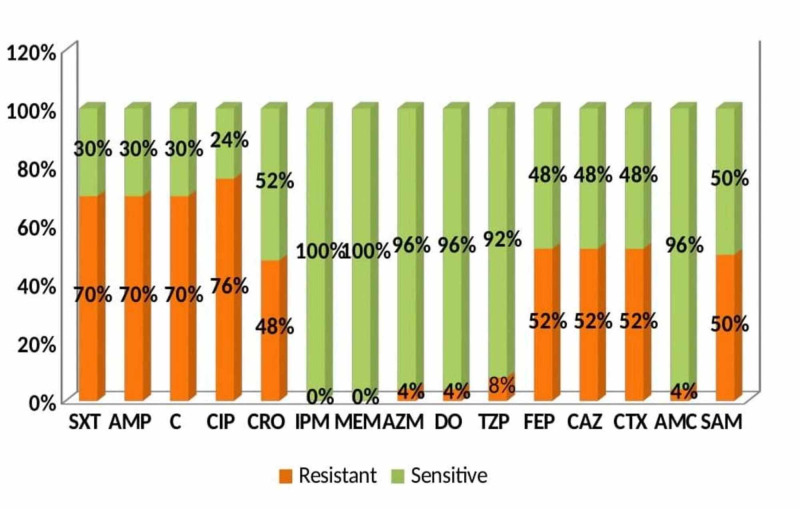
Antimicrobial Susceptibility Pattern of Salmonella Typhi and Salmonella Paratyphi SXT, Trimethoprim-sulfamethoxazole; AMP, ampicillin; C, chloramphenicol; CIP, ciprofloxacin; CRO, ceftriaxone; IPM, imipenem; MEM, meropenem; AZM, azithromycin; DO, doxycycline; TZP, piperacillin-tazobactam; FEP, cefepime; CAZ, ceftazidime; CTX, cefotaxime; AMC, co-amoxiclav; SAM, ampicillin-sulbactam

Among the first-line drugs (ampicillin, chloramphenicol, and trimethoprim-sulfamethoxazole), 70% of strains were resistant, and only 30% strains were sensitive to them. Among the cephalosporins, 52% strains were sensitive to ceftriaxone, and 48% strains were sensitive to ceftazidime, cefotaxime, and cefepime. Ciprofloxacin resistance was noted in 76% of strains. Only 50% of strains were sensitive to ampicillin-sulbactam, and 92% of strains were sensitive to piperacillin-tazobactam. All the strains were 100% sensitive to imipenem and meropenem; 96% of strains were sensitive to co-amoxiclav, doxycycline, and azithromycin. The frequency of MDR *Salmonella* was 16%, and XDR *Salmonella* was 54%. Figure 2 shows the antimicrobial susceptibility pattern of *Salmonella typhi* and *Salmonella paratyphi*.

## Discussion

Enteric fever is a common disease in developing countries due to several factors such as inadequate healthcare systems and poor sanitation [15]. Antibiotic resistance has become an important concern in treating the disease. *Salmonella *strains resistant to first-line antibiotics and third-generation cephalosporins have been isolated in Asia. The most common MDR haplotype of *Salmonella typhi *is H58, and it is spreading worldwide [10].

In our study, 94% of the *Salmonella* strains were *Salmonella typhi*, and only 6% were *Salmonella paratyphi A*. In another study, *Salmonella typhi *accounted for 84.5%, and *Salmonella paratyphi A* for 15.1% of the *Salmonella* strains. *Salmonella paratyphi B *and* C *were reported from only one case each, respectively [16]. In our study, most (68%) of patients with positive cultures were males. Similarly, Iyer et al. reported that 53.2% of the typhoidal cases were in males [16]. The majority (60%) of the patients with positive cultures were children with their ages ranging from 1 to 10 years followed by 11 to 20 years (26%) in our study. A study showed 61.9% of cases of *Salmonella* in five to 15 years of age and 23.4% cases in two to five years of age [16]. In our study, 62% of positive cases were reported from June to September 2019, 18% cases from March 2019 to May 2019, and 10% cases from October 2019 to February 2020. Similarly, most of the cases (36.4%) were reported from June to September in a study [16].

In our study, 70% of strains were resistant to ampicillin, chloramphenicol, and trimethoprim-sulfamethoxazole. Among the cephalosporins, 52% strains were sensitive to ceftriaxone, and 48% strains were sensitive to ceftazidime, cefotaxime, and cefepime. Ciprofloxacin resistance was noted in 76% of strains. Only 50% of strains were sensitive to ampicillin-sulbactam, and 92% of strains were sensitive to piperacillin-tazobactam. All the strains were 100% sensitive to imipenem and meropenem; 96% of strains were sensitive to co-amoxiclav, doxycycline, and azithromycin. The frequency of MDR *Salmonella *was 16%, and XDR *Salmonella* was 54%.

In a report by Chatham-Stephens et al. in the United States, *Salmonella *species causing enteric fever were isolated from 3538 blood cultures. Out of these, 65% were resistant to fluoroquinolones and 12% were MDR; 244 culture-positive patients had traveled to Pakistan, out of which 79% were fluoroquinolone-resistant and 50% were MDR [12]. In a study by Iyer et al., 100% of strains were sensitive to ceftriaxone and azithromycin [16]. In another study done in Shifa International Hospital, Islamabad, the frequency of MDR and XDR strains was 14% and 4.01%, respectively. A study conducted in India showed that all the typhoidal strains of *Salmonella *were sensitive to ceftriaxone and azithromycin. Ninety-four strains were also sensitive to chloramphenicol, and only 3.6% were sensitive to ofloxacin [17].

Laghari et al. reported 81.6% and 100% ampicillin-sensitive *Salmonella typhi *and *Salmonella paratyphi*, respectively, from Jamshoro, Southern Pakistan. Cotrimoxazole was sensitive in 96.4% *Salmonella typhi* and 100% *Salmonella paratyphi A*. Among the cephalosporins, 65.8% of the *Salmonella typhi* and 96.1% of *Salmonella paratyphi A *were ceftriaxone-sensitive. Ninety percent of *Salmonella typhi* and 94.2% of *Salmonella paratyphi A* were sensitive to cefotaxime. Ciprofloxacin was sensitive in 50.1% *Salmonella typhi* and 67.3% *Salmonella paratyphi A.* Imipenem showed sensitive zones in 87.8% *Salmonella typhi *and 96.1% *Salmonella paratyphi A.* Azithromycin was sensitive in 94.6% and 100% *Salmonella typhi* and *Salmonella paratyphi A*, respectively. The prevalence of MDR and XDR *Salmonella typhi* and *Salmonella paratyphi *was 2.6% and 0.9% [18]. Another study in Pakistan reported that the frequency of MDR *Salmonella typhi* was 76% and *Salmonella paratyphi *was 34%. The frequency of XDR *Salmonella typhi *was 48% [19].

In a study conducted in India, ampicillin resistance was noted in 29.47% *Salmonella typhi*, co-trimoxazole resistance in 17.89% strains, and chloramphenicol resistance in 28.42 strains. All the strains were sensitive to imipenem [15]. Another study done in India reported that all the *Salmonella typhi *strains were sensitive* *to imipenem, and 97.67% of strains were sensitive to meropenem. Eighty-three percent of *Salmonella typhi *were sensitive to ampicillin-sulbactam, 91% sensitive to ceftazidime, 90.6% sensitive to ceftriaxone, 57.5% sensitive to ciprofloxacin, and 57.5% sensitive to cotrimoxazole. The MDR *Salmonella typhi *were present in 44% of the cases [20].

The management of drug-resistant typhoidal strains of *Salmonella* has become a great challenge in Pakistan. The policies should be implemented at the government level to combat the rising burden of enteric fever. In addition, antimicrobial surveillance is poor, and antibiotic stewardship policies should be scaled-up. The use of antibiotics should be kept to a minimum. Antibiotics should not be available over the counter without the prescription of the doctor. Carbapenems and azithromycin should not be prescribed routinely unless the strain is resistant to first-line drugs, ciprofloxacin, and ceftriaxone on the antibiotic susceptibility report. However, the study has some limitations. A multicenter study should be conducted with a larger sample size. The antibiotic sensitivity testing should be done using the minimum inhibitory concentration (MIC).

## Conclusions

The increasing frequency of MDR and XDR *Salmonella *species in Pakistan is a major concern. A significant percentage of the typhoidal strains of *Salmonella* is resistant to first-line (16%) and second-line (54%) antibiotics. Carbapenems and azithromycin are the last resort of therapy in such cases.

## Appendices

RESEARCH PARTICIPANT CONSENT FORM

FOR RESEARCH PROJECT

Serial No: _______________ Date: ________________

Study Center: Sharif Medical and Dental College, Lahore

PART I: Information Sheet

Introduction: The participants will be explained in a language that they can understand easily. After a brief introduction, the participants are explained that they are invited to participate in the research. They will be informed that they may talk to anyone they feel comfortable with about the research and that they can take time to reflect on whether they want to participate or not. Assure the participant that if they do not understand some of the words or concepts that you will take time to explain to them as you go along and that they can ask questions now or later.

Purpose: The study aimed to determine the trends in antimicrobial resistance (AMR) of typhoidal strains of *Salmonella* in a tertiary care hospital in Pakistan. Due to the extensive overuse of antimicrobials, the world is moving toward a pre-antibiotic era. The emergence and transmission of antibiotic-resistant *Salmonella *species are a global threat and serious concern in developing countries such as Pakistan. This study will help in the scaling-up of policies at the government level in Pakistan and other countries with a high prevalence of disease to combat the rising burden of enteric fever.

Procedure: This study will require 10 ml blood sample from each patient through venipuncture using aseptic measures. All the required particulars of the patient will be recorded on the proforma sheet. Each sample will be processed by culture, gram staining, antibiotic susceptibility testing, and biochemical tests in the laboratory.

Voluntary Participation: Your participation in the study is entirely voluntary. Whether you choose to participate or not, all the services you receive at this hospital will continue in the same manner.

Time: Three to five minutes will be required for every patient to participate in this study.

Possible benefits: To diagnose enteric fever and determine the antibiotic susceptibility pattern of Salmonella.

Possible risks or discomfort: Venipuncture is a reasonably safe medical technique. However, bruising, hematoma formation and vasovagal syncope can occur in a few persons. The healthcare provider will try his/her best to decrease the chances of these complications but if any one of these occurs unexpectedly, you will be provided with immediate adequate medical care.

Financial Consideration: There will be no financial compensation for participation in the study. The financial benefit gained is free of cost testing for the participant.

Confidentiality: Your identity will not be disclosed, and all the records will be kept confidential. The result of the study including laboratory or any other data may be published.

Sharing the results: The results of your tests will be shared with you.

Withdrawal from Participation: You will have the right to refuse to participate in the study even if the consent form is signed. It will not affect your treatment or rights as a patient here.

Who to Contact: Provide the name and contact information of someone who is involved, informed, and accessible (a local person who can actually be contacted).

This proposal has been reviewed and approved by the institutional review board (IRB), which is a committee whose task is to make sure that research participants are protected from harm.

PART II: Certificate of Consent

I have read the foregoing information, or it has been read to me. I have had the opportunity to ask questions about it, and any questions that I have asked have been answered to my satisfaction. I consent voluntarily to participate as a participant in this research.

Name of Participant: _________________________ Age: ____________

Signature of Participant: ___________________

If illiterate

If the research participant is illiterate, a literate witness will sign (witness is selected by the participant and has no connection to the research team). Participants who are illiterate should include their thumbprint as well.

I have witnessed the accurate reading of the consent form to the potential participant, and the individual has had the opportunity to ask questions. I confirm that the individual has given consent freely.

Name of Witness Participant: _____________________

Signature of Witness: ______________________

Thumbprint of Participant: ______________________
